# Genome-wide profiling of RNA editing sites in sheep

**DOI:** 10.1186/s40104-019-0331-z

**Published:** 2019-03-12

**Authors:** Yuanyuan Zhang, Deping Han, Xianggui Dong, Jiankui Wang, Jianfei Chen, Yanzhu Yao, Hesham Y. A. Darwish, Wansheng Liu, Xuemei Deng

**Affiliations:** 1 Key Laboratory of Animal Genetics, Breeding and Reproduction of the Ministry of Agriculture & Beijing Key Laboratory of Animal Genetic Improvement, China Agricultural University, Beijing, 100193 China; 2 Department of Animal Science, Pennsylvania State University, University Park, PA 16802 USA; 3 Animal Production Research Institute, Agricultural Research Center, Ministry of Agriculture and Land Reclamation, Giza, 12618 Egypt

**Keywords:** DNA resequencing, RNA-DNA differences, RNA editing, RNA-seq, Sheep

## Abstract

**Background:**

The widely observed RNA-DNA differences (RDDs) have been found to be due to nucleotide alteration by RNA editing. Canonical RNA editing (i.e., A-to-I and C-to-U editing) mediated by the adenosine deaminases acting on RNA (ADAR) family and apolipoprotein B mRNA editing catalytic polypeptide-like (APOBEC) family during the transcriptional process is considered common and essential for the development of an individual. To date, an increasing number of RNA editing sites have been reported in human, rodents, and some farm animals; however, genome-wide detection of RNA editing events in sheep has not been reported. The aim of this study was to identify RNA editing events in sheep by comparing the RNA-seq and DNA-seq data from three biological replicates of the kidney and spleen tissues.

**Results:**

A total of 607 and 994 common edited sites within the three biological replicates were identified in the ovine kidney and spleen, respectively. Many of the RDDs were specific to an individual. The RNA editing-related genes identified in the present study might be evolved for specific biological functions in sheep, such as structural constituent of the cytoskeleton and microtubule-based processes. Furthermore, the edited sites found in the ovine *BLCAP* and *NEIL1* genes are in line with those in previous reports on the porcine and human homologs, suggesting the existence of evolutionarily conserved RNA editing sites and they may play an important role in the structure and function of genes.

**Conclusions:**

Our study is the first to investigate RNA editing events in sheep. We screened out 607 and 994 RNA editing sites in three biological replicates of the ovine kidney and spleen and annotated 164 and 247 genes in the kidney and spleen, respectively. The gene function and conservation analysis of these RNA editing-related genes suggest that RNA editing is associated with important gene function in sheep. The putative functionally important RNA editing sites reported in the present study will help future studies on the relationship between these edited sites and the genetic traits in sheep.

**Electronic supplementary material:**

The online version of this article (10.1186/s40104-019-0331-z) contains supplementary material, which is available to authorized users.

## Background

According to the central dogma, it is assumed that the sequence of mRNA truthfully reflects that of the DNA template. However, we know that the RNA sequences are not coded one-to-one by their corresponding DNA sequences. RNA modifications during co- and post-transcriptional processes contribute to the complexity of alternative splicing, and sequences and structures of the mature RNA molecules. One of the most important mechanisms is RNA editing, which results in RNA–DNA differences (RDDs), e.g., codon changes leading to protein variants [1]. It has been illustrated that the base modification at the RNA level plays an important role in post transcriptional regulation to enhance the complexity of transcripts and alter the function of genes.

The most commonly observed RNA editing event in human is A-to-I editing (normally interpreted as guanosine during transcription or by sequencing enzymes, recognized as A-to-G). The genes of the adenosine deaminases acting on RNA (ADAR) family were investigated to mediate the A-to-I editing process by binding the double-stranded (ds) RNA secondary structure [2]. Another widely described RNA editing base modification type in mammals is C-to-U conversion mediated by catalytic deaminase, apobec-1, and apobec-1 complementation factor (ACF) in apolipoprotein B mRNA editing catalytic polypeptide-like (APOBEC) family. The C-to-U conversion type of editing is relatively rare in the human transcriptome [3]. Both A-to-I and C-to-U events are considered as canonical RNA editing types, whereas the other types of RNA editing are generally considered as false positive detections in human genome-wide scanning studies [4–6]. However, there are some reports on the importance of the non-canonical editing types [7, 8].

According to the Rigorously Annotated Database of A-to-I RNA Editing (RADAR) database [9], only a few dozen human RNA editing targets that change amino acids in non-repetitive regions have been identified. An example is the initially detected RNA editing event, glutamate ionotropic receptor AMPA type subunit 2 (GluR-2) Q/R site resulting amino acid substitution, converting glutamine codon into arginine codon [10]. To date, the development of high-throughput sequencing technology such as next generation sequencing methods have facilitated the discovery of RNA editing events and its functional mechanisms in different organisms. In addition to studies on human tissues, several RNA editing sites have been reported in farm animals such as pig [11] and chicken [12].

To the best of our knowledge, there is no report on genome-wide scanning of RNA editing sites in sheep. In the present study, for the first time, we applied DNA and RNA resequencing data to identify potential editing events in sheep. We restricted our search for RNA editing sites only to homogenous genomic DNA (gDNA) sequences, but heterogenous RNA sequences as putative RDDs were also included to minimize false positives. In addition, crucial thresholds were set for the detection of RDDs for RNA and DNA mapping and genotype quality in order to eliminate uncertain reads and single-nucleotide variants (SNVs). Furthermore, we excluded the RDDs that are present on repetitive regions, which may result in mis-mapped reads and variant call errors [13, 14]. Overall, this study aims to explore reliable RNA editing events in sheep and provide more information for RNA-editing database of mammals.

## Methods

### Sample collection

The kidney and spleen tissues, and blood samples were obtained from three adult (2 years old) male Lanping sheep (a Chinese indigenous sheep breed) from the same population in Yunnan Province of China. These animals were unrelated according to their pedigree records. DNA was extracted from blood samples with the routine phenol–chloroform extraction method. The total RNA was extracted and purified using the RNA Sample Total Kit (Qiagen, German). The quality of gDNA and RNA was evaluated using NanoDrop 2000 (Thermo Fisher Scientific) and by agarose gel electrophoresis (1.2% agarose gel).

### DNA/RNA sequencing

For each DNA sample, a whole-genome sequencing library was built using the Illumina TruSeq DNA Sample Preparation Kit (Illumina, Inc., San Diego, CA, USA) with an insert size of ~ 350 bp. The libraries were sequenced on the Illumina HiSeq 2000 platform. The paired-end reads of 100 bp were generated for each fragment.

The total RNA from each sample was used as input for the TruSeq RNA Prep Kit (Illumina, Inc.) and by means of indexed adapters; a sequencing library was created according to the manufacturer’s instructions. RNA sequencing was conducted on the Illumina HiSeq 2500 platform, resulting in paired-end 100-bp reads. The insert length of the RNA-seq library ranged from 300 to 400 bp. The DNA- and RNA-seq data have been deposited to the National Center for Biotechnology Information (NCBI) SRA Database (accession number: SRP133430).

### Mapping and variant calling strategies

The raw reads of both DNA- and RNA-seq data were first checked using the FastQC tool [15] and trimmed using Trimmomatic [16]. First six bases of each read were discarded to avoid artificial mismatches derived from random-hexamer priming, adaptors, and low-quality nucleotides (quality < 30) were also removed. The resulting clean reads were obtained and prepared for alignment against sheep reference genomic sequences (OARv4.0).

For the RNA-seq data, the reads were mapped to the sheep reference genome using the two-pass mapping strategy in STAR (v2.6) [17], and only uniquely mapped reads were extracted based on the mapping quality (MAPQ = 255). We then used the GATK ReassignOneMappingQuality read filter of GATK (v4.0.12.0) [18] to reassign map quality scores (MAPQ) of all unique alignments from 255 to the default value of 60 in GATK (v4.0.12.0) [18]. The BWA (v0.7.17) “mem” method [13] with default parameters was used to align DNA reads against the sheep reference genome (OARv4.0). The polymerase chain reaction duplicates were removed from the resulting DNA and RNA mapped bam files using the MarkDuplicates tool from GATK (v4.0.12.0) [18]. The results from the two mapping procedures were merged into a single BAM file.

Joint variant calling was conducted on the BAM file using Samtools/Bcftools [19, 20] to construct a preliminary file of known variants, which would be applied for the GATK indel realignment and base quality recalibration steps. HaplotypeCaller was then used for variant calling across all DNA and RNA alignments using the options stand_call_conf 10 and stand_emit_conf 10. According to the previous literatures, a batch of filters should be conducted to remove false positive sites resulting from sequencing and mapping bias [5, 21]. In the variant filtering step, indels were removed and only biallelic SNPs were retained for the further filtering steps. Variants that failed one of the following filters were removed: 1) alternative variants that were supported by at least two reads, 2) mapping quality score of ≥20, 3) base quality score of ≥95, and 4) strandness Fisher test value of ≤30.

### Detection of putative RNA-DNA differences

In the detection of RNA–DNA differences, only positions supported by at least four genomic reads and completely homozygous were retained. The following types of mismatches were also discarded according to a previous study [5]: 1) supported by < 10 RNA-seq reads, 2) intronic mismatches located within 4 bp of a known splice junction, 3) edit ratio (fraction of edited vs. total reads) < 10% or > 95%, extremely low or high edit ratio could result from low coverage of genomic reads or sequencing error. To reduce false positives caused by sample artefact, RDDs detected in all the three biological replicates were kept and regarded as common RNA editing sites in the tissue. These types of restricted editing sites were collected and used for the further annotation.

Due to our RNA-seq data are not strandness specific, we utilized the gene annotation file (ftp://ftp.ncbi.nlm.nih.gov/genomes/Ovis_aries/GFF/) to extract the RDDs located in the transcriptional regions, and then identified their types with the help of the strand information of each transcript.

### Validation of RNA-DNA differences

To validate the homozygous/heterozygous status of the putative RDDs, we used PCR to amplify RDDs on 13 putative editing sites. The PCR products of DNA and cDNA samples were sequenced by Sanger sequencing. Primers were designed using the NCBI Primer-BLAST tools (https://www.ncbi.nlm.nih.gov/tools/primer-blast/). The primer sequences are listed in Additional file 1: Table S3. The PCR reaction mixture of volume 20 μL was prepared with 1× PCR mix, 20 ng of genomic (or 5 ng of cDNA) template, and 500 nmol/L each of forward and reverse primers.

### Differential expression analysis and gene annotation

Transcriptome quantification and differential expression analyses were performed using Cuffdiff [22]. ANNOVAR [23] was used to annotate the editing sites using the GFF3 files (ftp://ftp.ncbi.nlm.nih.gov/genomes/Ovis_aries/GFF/). The genes with mismatches located in the exonic regions were extracted for the functional enrichment analysis. Gene Ontology annotation were computed using the DAVID web tool [24].

To analyze the conservation of upstream and downstream sequences of an editing site, alignment of genomic sequences among different species was conducted using the alignment tool, which is available on Ensembl (https://www.ensembl.org/index.html).

## Results

### Read mapping and variant calling

The trimmed reads and mapping alignments of each sample are listed in Table 1.

**Table 1:** Alignment of clean reads of DNA and RNA samples

Alignment	DNA		Kidney RNA		Spleen RNA	
	Raw reads	MQ > 20	Raw reads	Uniquely mapped	Raw reads	Uniquely mapped
IND1	228,494,214	85.81%	84,918,643	52.63%	71,544,583	56.74%
IND2	310,038,163	87.75%	78,384,278	48.53%	68,023,085	54.89%
IND3	227,778,931	86.23%	82,092,796	54.92%	73,439,711	57.89%

After all the DNA reads of the three individuals were pooled, we estimated the average coverage of genomic sequences to be 26×. Accurate identification of variants from RNA and DNA of the same individual is the most important step in identifying RDDs. To avoid miscalled SNVs owing to sequencing errors and misalignments, we applied stringent filters (details are mentioned in methods). Transcriptome-wide screening revealed 84,361 and 97,295 high-quality heterozygous SNVs in the kidney and spleen tissues of sheep, respectively.

### Identification of RNA editing

In the present study, SNV sites that were homozygous at the DNA level and heterozygous at the transcriptome level were extracted as putative RNA editing sites. After comparing the DNA-seq and RNA-seq reads generated from the same individuals and applying additional filters for removing RDDs in the splicing region, a total of 18,644 and 25,407 RDDs in the kidney and spleen were obtained, respectively. In the three kidney tissues replicates, 7,869, 6,036, and 7,779 RDDs were retained, respectively. Furthermore, 10,391, 9,253, and 10,702 RDDs were obtained from the three spleen tissue samples (Additional file 1: Figure S1). Finally, 607 common RDDs in the kidney and 994 common RDDs in the spleen were extracted as the reliable editing sites. All the putative RDDs from the three sheep individuals were listed in Additional files 2, 3 and 4.

We validated 13 RDDs by Sanger sequencing method, 9 of the 13 sites were confirmed as the true edited sites. The sites on chr11:41753132 were considered false positives by comparing their gDNA and cDNA sequencing chromatograms. At the two predicted editing sites of *CARD19*, two overlapping peaks were observed at the cDNA sequencing chromatograms; these peaks might result from incomplete RNA editing at these sites for all mRNA molecules in the kidney and spleen. However, the DNA sequencing of *CARD19* failed, and the editing status of the two predicted sites was set as “Not sure”. The chromatogram traces of gDNA and cDNA (extracted from the kidney and spleen) are shown in Additional file 1: Table S1.

**Fig. 1:**
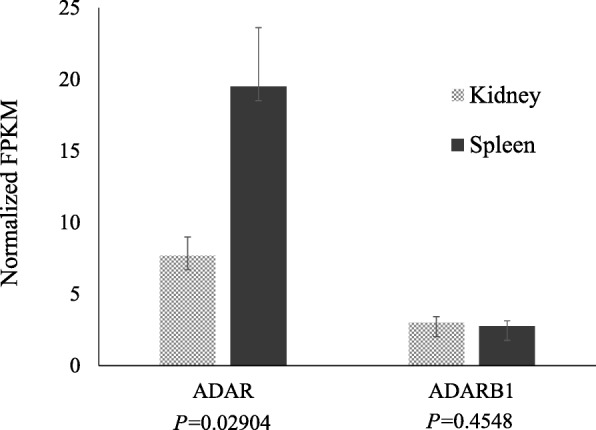
Normalized expression levels of ADAR and ADARB1 between the kidney and spleen tissues. Expression between the tissues was calculated by tissue-scale normalization. Pairwise *t*-test was used to determine the differences in the expression levels between the spleen and kidney. The *ADAR* expression was significantly (*P* < 0.05) higher in the spleen than in the kidney, but the *ADARB1* expression was not significantly different between the spleen and kidney

The number of RDDs found in the spleen was marginally higher than that in the kidney, likely because the expression of ADAR was significantly (*P*< 0.01) higher in the spleen than in the kidney (Fig. 1). The adjusted fragments per kilo base per million (FPKM) sequenced read values in the spleen and kidney were 19.52 and 7.71, respectively. The expression of ADARB1 (adenosine deaminase, RNA specific B1, also known as ADAR2) was not significantly different between the kidney and spleen. The ADAR family comprises ADAR (also known as ADAR1), ADARB1, and ADARB2 (adenosine deaminase, RNA specific B2, also known as ADAR3). In human, ADAR and ADARB1 are expressed in almost all the tissues, and they have the ability to convert adenosine to inosine in double-stranded RNAs [25–27]. ADARB2 was not found in our sheep RNA-seq data, and it is reported to be expressed only in human brain without a known editing activity.

### The genomic location of RDDs

To understand the functional importance of the RDDs detected in our study, we clustered these sites according to their locations to the nearest known genes. In total, 607 and 994 RDDs in the spleen and kidney were found in all the three biological replicates simultaneously and were considered as common RNA editing sites.

**Fig. 2:**
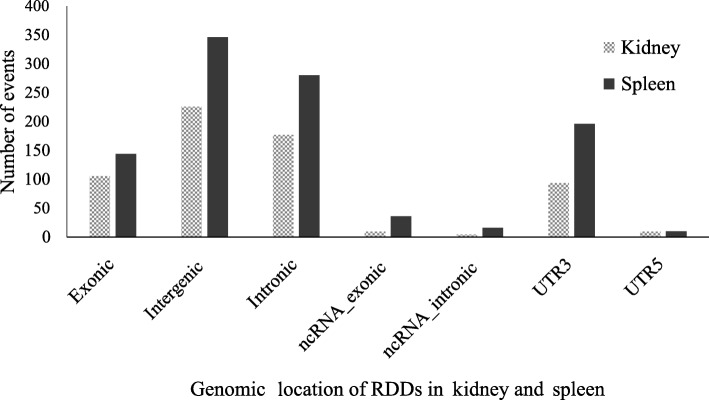
Distribution of the locations of the common RDDs in kidney and spleen. Genomic location of each RDD was classified depending on the proximity to the nearest known gene. Summary of the locations of RDDs from 607 common RDDs in kidney and 994 common RDDs in spleen

Most of the common RNA editing sites in the spleen and kidney were annotated on exonic, intronic, and intergenic mismatches. One third of the RDDs in the kidney (226/607) and spleen (346/994) was found in the intergenic regions; the proportion of exonic DNA-RNA mismatches was both close to 15% in the kidney (106/607) and spleen (144/994). In the intronic regions, the mismatches in the kidney (177/607) and spleen (346/994) were made up of 27–28% RDDs in total. Especially, additional 15 and 52 mismatches were detected in ncRNAs in the kidney and spleen, respectively (Fig. 2). In addition to the common RNA editing sites, a large range of individual-specific RDDs were detected in either the spleen or kidney tissue, respectively. More than half of these individual-specific RDDs were annotated within the protein-coding and UTR regions. Irrespective of the tissues, RDDs seemed to be frequent in the UTR and protein-coding regions. In the present study, we performed further analysis on the common RNA editing sites.

### Types of RDDs

**Fig. 3:**
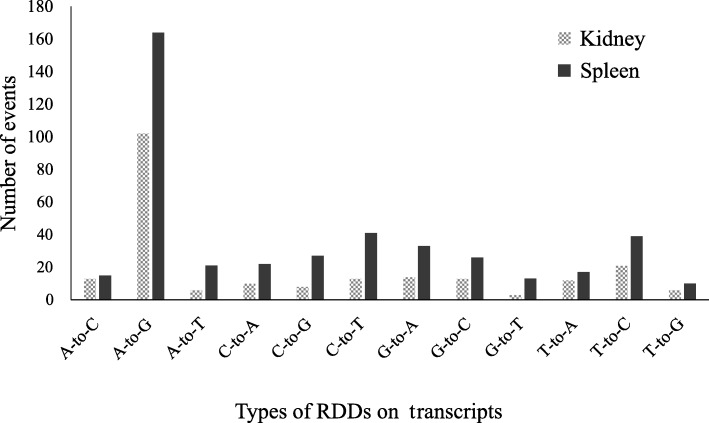
Types of observed RDDs on the transcripts. 221 and 428 common RDDs were found locating on the transcriptional regions. Among the 12 types of RDDs, the most frequent event was A-to-G

Because our RNA-seq data are not strand-specific, the types of RDDs can be wrong if the types of mismatches identified in the variant detection analysis were directly used. We utilized the gene annotation file to extract the RDDs located in the transcriptional regions, and then identified their types with the help of the strand information of each transcript. The analyses of RDD types indicated that A-to-G type was the most frequent (38–46%) type of mismatches between DNA and RNA in transcriptional regions (Fig. 3). The second frequent type in transcriptional regions was T-to-C mismatches, which made up ~ 9% of the mismatches in both the kidney and spleen. The number of A-to-G mismatch was higher in the spleen (164) than in the kidney (102). This can be attributed to the fact that A-to-G editing is mainly mediated by ADAR, and the expression of which was significantly higher in the spleen than in the kidney.

### Gene annotation of the common RDDs in the kidney and spleen

In total, 164 and 247 genes were annotated to the kidney and spleen RDDs (excluding intergenic RDDs), respectively. Eighty-three genes were found annotated in both the kidney and spleen RDDs. In the kidney, 44 RDDs (21 genes) in the kidney and 63 RDDs (33 genes) in the spleen were found to be nonsynonymous mutations resulting in amino acid changes or stop–gain mutations (Additional file 1: Tables S4 and S5). The enrichment analysis indicated that among the 164 and 247 genes annotated to the RDDs in the kidney and spleen, five genes related to the peroxisome term (GO:0005777) were slightly overrepresented, and the BH adjusted *P* value was 4.7e-2. Other RNA editing-related genes identified in the present study might be evolved in specific biological functions of sheep, such as structural constituent of the cytoskeleton and microtubule-based process, even though the BH adjusted *P* value was not significant. RNA editing related genes that categoried to GO terms were listed in Additional file 1: Tables S6 and S7.

**Fig. 4:**
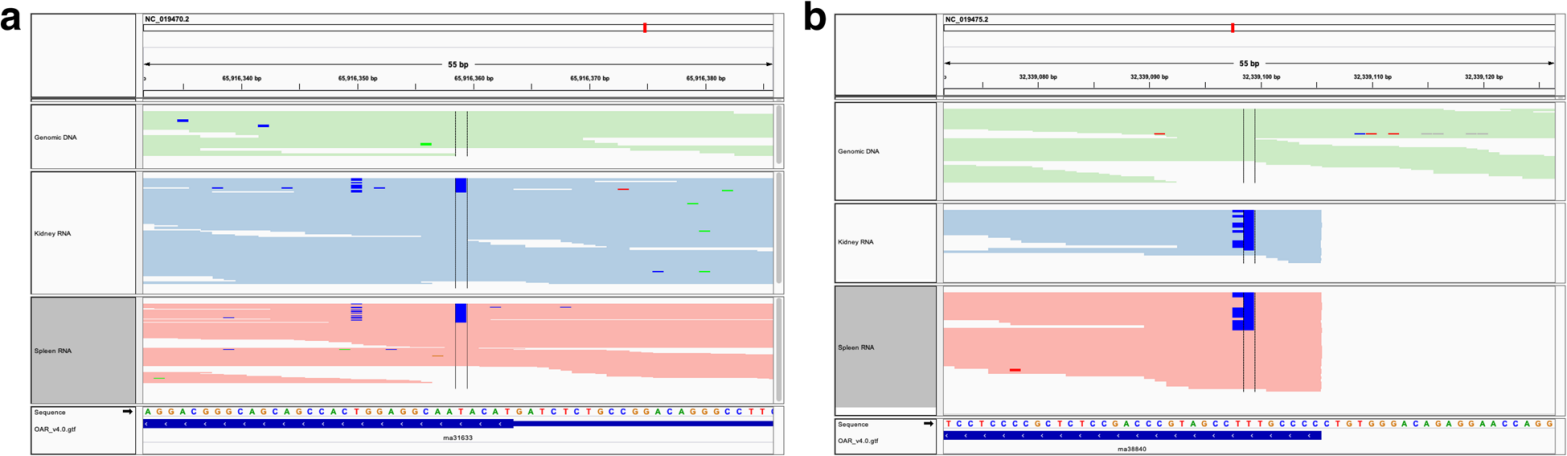
IGV screenshot for RNA editing sites in *BLCAP* (**a**) and *BEIL1* (**b**). The two IGV screenshots showing the alignments of genomic and RNA reads in *BLCAP* (**a**) and *BEIL1* (**b**). The two editing sites (NC_019470.2:65916359 and NC_019475.2:32339099) were at the center lines. From top to bottom, the tracks are as follows: genomic DNA reads, RNA reads in Kidney, RNA reads in Spleen, reference sequences and transcripts

Interestingly, exploration of the human RNA editing database RADAR [9] (http://rnaedit.com/download/) and the previously reported RNA editing sites, revealed that bladder cancer associated protein (*BLCAP*) and Nei endonuclease VIII-like 1 (*NEIL1*) have non-repetitive RDD sites.The type and location of the two editing sites on genomic reads and RNA reads were shown by IGV tools in Fig. 4. Further, 2,576,459 RNA editing events were recorded in the database RADAR, and 95.56% of the events were located on the ALU elements in human; 103 and 137 RNA editing-related genes in the kidney and spleen were found in the RADAR database. Among these edited genes, two edited sites in *BLCAP* and *NEIL1* were also found in previous studies in chicken [12] and pig [28].

In the present study, the editing site (amino acid change: Y/C) on exon 1 of *BLCAP* was found in both the kidney and spleen, and the K/R editing site on exon 6 of *NEIL1* was found in the spleen of the three adult male sheep.

To investigate the conservation of regions of these RDDs between sheep and other mammal species, multiple sequence alignments of *BLCAP* and *NEIL1* genomic sequences were performed using the Ensembl online alignment tool. The edited sites detected in *BLCAP* and *NEIL1* were both located in conserved genomic regions with 100% sequence similarity among these species (Additional file 1: Figures S2 and S3).

## Discussion

RNA editing is a widespread phenomenon occurring during the co- and post-transcriptional processes. Most of the known editing sites in humans are found in non-coding regions, especially in repetitive genomic structures (for instance, the ALU elements), and in the UTRs of genes [5, 29, 30]. In our study, we used the DNA- and RNA-seq data to detect 18,644 and 25,407 RDDs in sheep kidney and spleen, however, most of the detected RDDs were individual-specific. In order to control false positives, strict detection filters were applied, such as, modest RNA-seq coverage, high quality of mapped reads and high qulity of mapped alleles. The numbers of RNA editing sites are expected to be underestimated in the present study. Many sites are edited at a low frequency and the ability to be detect comes with the increase of sequencing depth [31]. We then focused on RDDs located on UTRs and protein coding regions, which are considered to be likely correlated with gene functions. There should be more number of editing sites in sheep than that identified in the present study, because we only focused on RDDs and used restrictive filtering parameters to minimize noise; thus, we provided some reliable RNA editing sites in sheep, but inevitably we missed some true sites.

### Types of RDDs

Generally, among all the RDD types, A-to-I and C-to-U are presumed to be canonical editing sites; the other 10 non-canonical editing sites were usually presumed to be false-positive results due to the current sequencing technology and analysis limitations [5].

Some studies have indicated that both canonical and non-canonical editing sites exist in the transcriptome [32]. The C-to-T editing mediated by the APOBEC family has been found to increase the number of C-to-T and G-to-A mismatches, because the C-to-U editing sites can produce C-to-T editing in the primary strand, whereas G-to-A editing occurs on the complementary strand [33]. Similarly, A-to-I editing might increase the A-to-G and T-to-C mismatches. Our transcriptomic data showed that *APOBEC1* is not expressed in the two tissues investigated, but *APOBEC2* and *APOBEC3F* were found to be expressed at a low level (Additional file 1: Table S2). Furthermore, we observed other non-canonical RNA editing sites, such as G-to-A and T-to-C in both the kidney and spleen. This can be attributed to the fact that our RNA-seq data were not strand-specific. Moreover, the type of a RDD has a high probability of being erroneously called to be a different type. Additionally, RDDs identified from RNA-seq reads covering or intersecting with known genes can be assumed to originate from these genes. Thus, the percentage of RDD type within known genes were estimated, however, RDDs in unannotated regions or in regions with bidirectional transcription were not taken into any categories. The results showed that A-to-G type was the most frequent (38–46%) type of mismatches between DNA and RNA in transcriptional regions. Future study with strand-specific RNA-seq is needed to confirm the non-canonical RNA editing sites identified in the present study.

### Tissue-specific edited sites might play important roles in regulating gene expression

The RNA editing events that result in changes in amino acid residues are prevalent regulation changes of phenotypes, and the widespread RNA editing events are known to play an important role in the diversity of the transcriptome by producing presumably more types of transcripts, while affecting only a few nucleotides [34]. In the present study, we identified 18,644 and 25,407 RDDs in the kidney and spleen of sheep, respectively. More than 90% RDDs showed tissue or individual specificity, indicating that the large proportion of physiological differences among individuals and tissues might be attributed to the diversity in the transcriptional and post-transcriptional processes. A previous study has also suggested that RDDs show cell or tissue specificity during development; they are probably not functionally important because of their rare occurrence within populations [27]. Conversely, common editing sites can be associated with certain advantages and hence are preserved during evolution [5]. RNA editing can alter gene expression by affecting the binding of small regulatory RNAs (microRNAs and siRNAs), creating alternative splice sites, and changing the sites in stop codons [35, 36]. Therefore, the editing sites might be important for the variation in gene expression and phenotypic features.

### Conserved edited sites in *BLCAP* and *NEIL1*

Within all the three biological replicates, 75 genes were found containing reliable RNA editing sites in sheep. Two edited sites in *BLCAP* and *NEIL1* are also reported in other species. Furthermore, the sequences near the edited sites in *BLCAP* and *NEIL1* have high similarities among mammal species (Additional file 1: Figures S2 and S3), suggesting that these editing events might be conserved in mammals.

*BLCAP*, a highly conserved gene with tumor-suppressor function in different carcinomas, was previously identified as an edited gene in the brain [37] and fat tissue of pig [11], and the same Y/C editing site is mapped in the key YXXQ motif in human. *BLCAP* with RNA editing at this site was found to lose its ability to inhibit signal transducer and activator of transcription 3 (STAT3), providing a clue that the Y/C editing site in BLCAP is associated with the function of BLCAP and the development of cancer [38]. Exon 6 K/R RDD found in the present study in the sheep *NEIL1* gene, which codes for a DNA repair enzyme, has also been reported in multiple tissues in human [39] and pig [11]. Additionally, it has been reported that the edited and unedited forms of the NEIL1 proteins have distinct enzymatic properties for glycosylase activity and lesion specificity, and the editing level can be regulated extracellularly by interferon [40].

These editing events occur in the protein coding region can directly alter the protein sequences and structures, and then affect the biological function. These results suggest a specific regulatory mechanism for gene function derived by RNA editing.

## Conclusions

To the best of our knowledge, our study is the first to detect RNA editing in sheep by comparing RNA-seq and DNA-seq data from three biological replicates of the kidney and spleen tissues. Under stringent sequence data filters, a total of 607 and 994 common RDDs were identified in the ovine kidney and spleen as the reliable RNA editing sites. Further,164 and 247 genes were annotated to the kidney and spleen RDDs. The amino acid conversion edited sites found in the ovine *BLCAP* and *NEIL1* are conserved in pig and human, suggesting that evolutionarily conserved RNA editing sites may be important for gene function and the development of an individual. Our study provides some putative functionally important RNA editing sites in sheep, and it is important to conduct further studies to reveal the functional changes in RNA editing event genes and their roles in economically beneficial traits and diseases in sheep.

## Additional files

Additional file 1: **Figure S1.** Shared RDDs of the Three Biological Replicates in the Kidney and Spleen. **Figure S2.** Conservation Analysis of *BLCAP*. **Figure S3.** Conservation Analysis of *NEIL1*. **Table S1.** Validation of RNA Editing Sites by Sanger Sequencing. **Table S2.** FPKM (mean ± SD) of *APOBEC3F* and *APOBEC2* in the Kidney and Spleen. **Table S3.** PCR Primers Used in Sanger Sequencing Validation. **Table S4.** Nonsynonymous Common Editing Sites in the Kidney of Sheep. **Table S5.** Nonsynonymous Common Editing Sites in the Spleen of sheep. **Table S6.** Functional Annotation Results of RNA Editing Genes in the Kidney of sheep. **Table S7.** Functional Annotation Results of RNA Editing Genes in the spleen of sheep. (DOCX 1050 kb)

Additional file 2: Putative RDDs in Sheep Individual1. (XLSX 1098 kb)

Additional file 3: Putative RDDs in Sheep Individual2. (XLSX 880 kb)

Additional file 4: Putative RDDs in Sheep Individual3. (XLSX 1115 kb)

## Abbreviations

ADAR: Adenosine deaminases acting on RNA
cDNA: Complementary DNA
ds: Double-stranded
FPKM: Fragments per kilo base per million
gDNA: Genomic DNA
GO: Gene ontology
KEGG: Kyoto Encyclopedia of Genes and Genomes
MAPQ: Map quality score
RADAR: Rigorously Annotated Database of A-to-I RNA Editing
RDD: RNA-DNA difference
SNP: Single nucleotide polymorphism
SNV: Single-nucleotide variant
UTR: Untranslated region

## References

[1] Gott JM, Emeson RB (2000). Functions and mechanisms of RNA editing. Annu Rev Genet.

[2] Slotkin W, Nishikura K (2013). Adenosine-to-inosine RNA editing and human disease. Genome Med.

[3] Rosenberg BR, Hamilton CE, Mwangi MM, Dewell S, Papavasiliou FN (2011). Transcriptome-wide sequencing reveals numerous APOBEC1 mRNA-editing targets in transcript 3’ UTRs. Nat Struct Mol Biol.

[4] Park E, Guo J, Shen S, Demirdjian L, Wu YN, Lin L (2017). Population and allelic variation of A-to-I RNA editing in human transcriptomes. Genome Biol.

[5] Zhang Q, Xiao X (2015). Genome sequence-independent identification of RNA editing sites. Nat Methods.

[6] Ramaswami G, Lin W, Piskol R, Tan MH, Davis C, Li JB (2012). Accurate identification of human Alu and non-Alu RNA editing sites. Nat Methods.

[7] Rubio MAT, Paris Z, Gaston KW, Fleming IMC, Sample P, Trotta CR (2013). Unusual noncanonical intron editing is important for tRNA splicing in Trypanosoma brucei. Mol Cell.

[8] Zheng Y, Ji B, Song R, Wang S, Li T, Zhang X (2016). Accurate detection for a wide range of mutation and editing sites of microRNAs from small RNA high-throughput sequencing profiles. Nucleic Acids Res.

[9] Ramaswami G, Li JB (2014). RADAR: a rigorously annotated database of A-to-I RNA editing. Nucleic Acids Res.

[10] Sommer B, Kohler M, Sprengel R, Seeburg PH (1991). RNA editing in brain controls a determinant of ion flow in glutamate-gated channels. Cell.

[11] Funkhouser SA, Steibel JP, Bates RO, Raney NE, Schenk D, Ernst CW (2017). Evidence for transcriptome-wide RNA editing among Sus scrofa PRE-1 SINE elements. BMC Genomics.

[12] Fresard L, Leroux S, Roux PF, Klopp C, Fabre S, Esquerre D (2015). Genome-wide characterization of RNA editing in chicken embryos reveals common features among vertebrates. PLoS One.

[13] Li H, Durbin R (2009). Fast and accurate short read alignment with burrows-wheeler transform. Bioinformatics.

[14] Robasky K, Lewis NE, Church GM (2014). The role of replicates for error mitigation in next-generation sequencing. Nat Rev Genet.

[15] Andrews S. FastQC- A quality control tool for high throughput sequence data. https://www.bioinformatics.babraham.ac.uk/projects/fastqc/.

[16] Bolger AM, Lohse M, Usadel B (2014). Trimmomatic: a flexible trimmer for Illumina sequence data. Bioinformatics.

[17] Dobin A, Davis CA, Schlesinger F, Drenkow J, Zaleski C, Jha S (2013). STAR: ultrafast universal RNA-seq aligner. Bioinformatics.

[18] DePristo MA, Banks E, Poplin R, Garimella KV, Maguire JR, Hartl C (2011). A framework for variation discovery and genotyping using next-generation DNA sequencing data. Nat Genet.

[19] Li H, Handsaker B, Wysoker A, Fennell T, Ruan J, Homer N (2009). The sequence alignment/map format and SAMtools. Bioinformatics.

[20] Li H (2011). A statistical framework for SNP calling, mutation discovery, association mapping and population genetical parameter estimation from sequencing data. Bioinformatics.

[21] Lee JH, Ang JK, Xiao X (2013). Analysis and design of RNA sequencing experiments for identifying RNA editing and other single-nucleotide variants. RNA.

[22] Trapnell C, Hendrickson DG, Sauvageau M, Goff L, Rinn JL, Pachter L (2013). Differential analysis of gene regulation at transcript resolution with RNA-seq. Nat Biotechnol.

[23] Wang K, Li M, Hakonarson H (2010). ANNOVAR: functional annotation of genetic variants from high-throughput sequencing data. Nucleic Acids Res.

[24] Huang d W, Sherman BT, Lempicki RA (2009). Systematic and integrative analysis of large gene lists using DAVID bioinformatics resources. Nat Protoc.

[25] Grice LF, Degnan BM (2015). The origin of the ADAR gene family and animal RNA editing. BMC Evol Biol.

[26] Terajima H, Yoshitane H, Ozaki H, Suzuki Y, Shimba S, Kuroda S (2017). ADARB1 catalyzes circadian A-to-I editing and regulates RNA rhythm. Nat Genet.

[27] Nishikura K (2010). Functions and regulation of RNA editing by ADAR deaminases. Annu Rev Biochem.

[28] Funkhouser SA, Steibel JP, Bates RO, Raney NE, Schenk D, Ernst CW (2017). Evidence for transcriptome-wide RNA editing among Sus scrofa PRE-1 SINE elements. BMC Genomics.

[29] Levanon EY, Eisenberg E, Yelin R, Nemzer S, Hallegger M, Shemesh R (2004). Systematic identification of abundant A-to-I editing sites in the human transcriptome. Nat Biotechnol.

[30] Li M, Wang IX, Li Y, Bruzel A, Richards AL, Toung JM (2011). Widespread RNA and DNA sequence differences in the human transcriptome. Science.

[31] Huntley MA, Lou M, Goldstein LD, Lawrence M, Dijkgraaf GJ, Kaminker JS (2016). Complex regulation of ADAR-mediated RNA-editing across tissues. BMC Genomics.

[32] Bahn JH, Lee JH, Li G, Greer C, Peng G, Xiao X (2012). Accurate identification of A-to-I RNA editing in human by transcriptome sequencing. Genome Res.

[33] Hamilton CE, Papavasiliou FN, Rosenberg BR (2010). Diverse functions for DNA and RNA editing in the immune system. RNA Biol.

[34] Goldstein B, Agranat-Tamir L, Light D, Ben-Naim Zgayer O, Fishman A, Lamm AT (2017). A-to-I RNA editing promotes developmental stage-specific gene and lncRNA expression. Genome Res.

[35] Jepson JE, Reenan RA (2008). RNA editing in regulating gene expression in the brain. Biochim Biophys Acta.

[36] Kawahara Y, Zinshteyn B, Sethupathy P, Iizasa H, Hatzigeorgiou AG, Nishikura K (2007). Redirection of silencing targets by adenosine-to-inosine editing of miRNAs. Science.

[37] Veno MT, Bramsen JB, Bendixen C, Panitz F, Holm IE, Ohman M (2012). Spatio-temporal regulation of ADAR editing during development in porcine neural tissues. RNA Biol.

[38] Chen W, He W, Cai H, Hu B, Zheng C, Ke X (2017). A-to-I RNA editing of BLCAP lost the inhibition to STAT3 activation in cervical cancer. Oncotarget.

[39] Picardi E, Manzari C, Mastropasqua F, Aiello I, D'Erchia AM, Pesole G (2015). Profiling RNA editing in human tissues: towards the inosinome atlas. Sci Rep.

[40] Yeo J, Goodman RA, Schirle NT, David SS, Beal PA (2010). RNA editing changes the lesion specificity for the DNA repair enzyme NEIL1. Proc Natl Acad Sci U S A.

