# Supplementary material for: Genome-wide profiling of RNA editing sites in sheep
Source: J Anim Sci Biotechnol. 2019 Mar 12;10:31. doi: 10.1186/s40104-019-0331-z (PMC6419479; doi:10.1186/s40104-019-0331-z)
Supplement: Supplementary file 1 — Figure S1. Shared RDDs of the Three Biological Replicates in the Kidney and Spleen. Figure S2. Conservation Analysis of BLCAP. Figure S3. Conservation Analysis of NEIL1. Table S1. Validation of RNA Editing Sites by Sanger Sequencing. Table S2. FPKM (mean ± SD) of APOBEC3F and APOBEC2 in the Kidney and Spleen. Table S3. PCR Primers Used in Sanger Sequencing Validation. Table S4. Nonsynonymous Common Editing Sites in the Kidney of Sheep. Table S5. Nonsynonymous Common Editing Sites in the Spleen of sheep. Table S6. Functional Annotation Results of RNA Editing Genes in the Kidney of sheep. Table S7. Functional Annotation Results of RNA Editing Genes in the spleen of sheep. (DOCX 1050 kb) [file 40104_2019_331_MOESM1_ESM.docx]

**ADDITIONAL FILE 1**

**Genome-wide Profiling RNA Editing Sites in Sheep**

Zhang et al., 2018


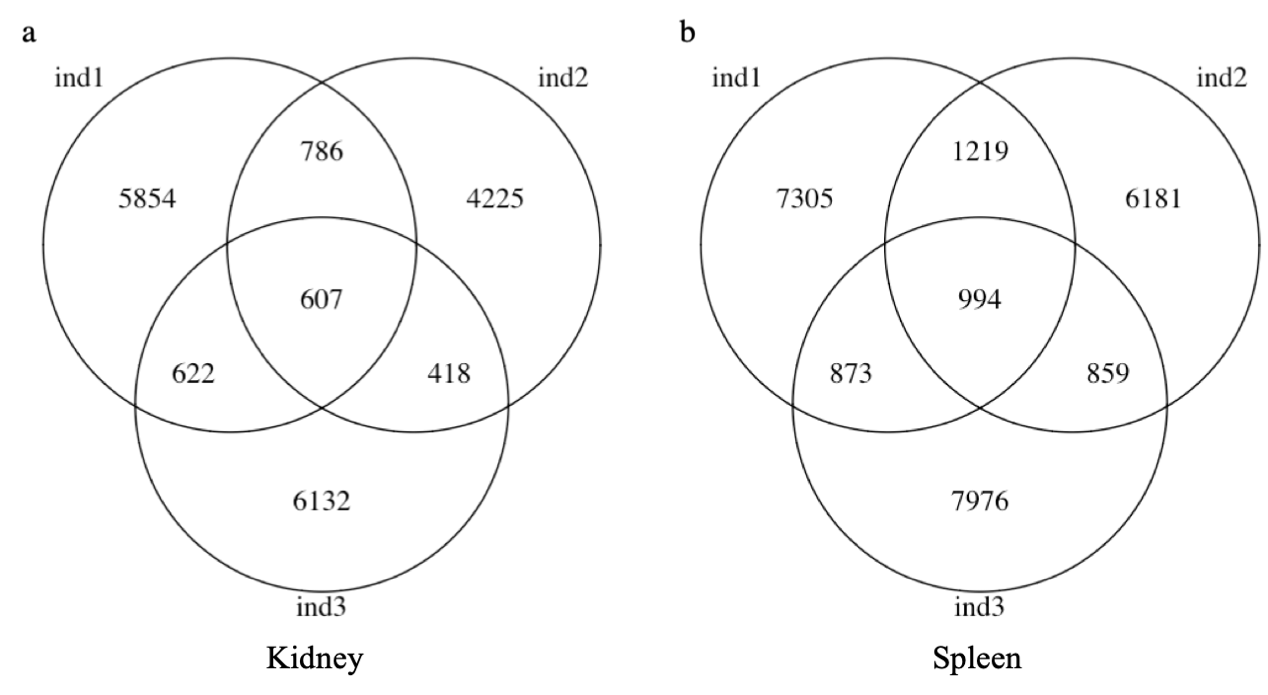


**Figure S1** Shared RDDs of the Three Individuals in Kidney (a) and Spleen (b). An RDD between two or three individuals was considered shared if it was identified at the same physical position.


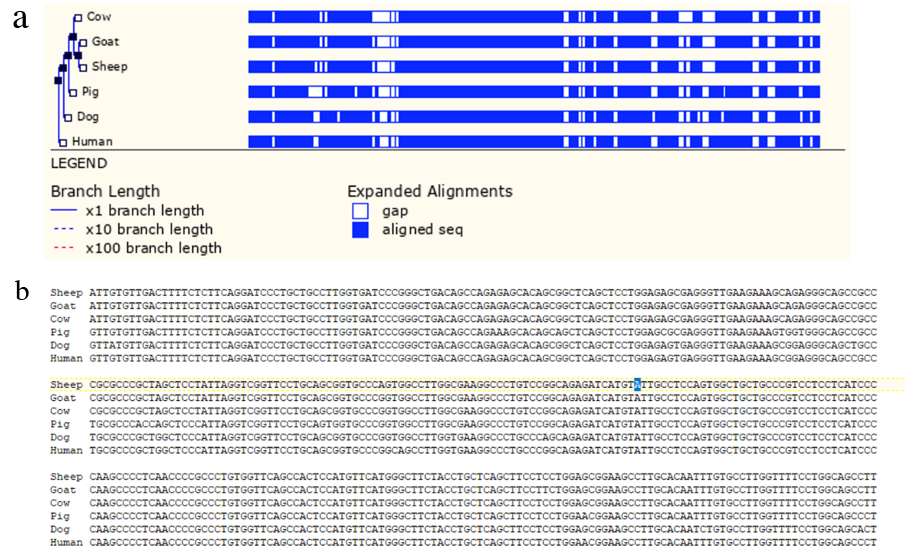


**Figure S2** Conservation Analysis of *BLCAP*. A: Comparison of the aligned regions of the genomic sequences among different species (Human, Dog, Pig, Cow, Goat and Sheep). B: Sequence alignment details near the edited sites of *BLCAP* (OAR13: 65916359A, marked in blue).


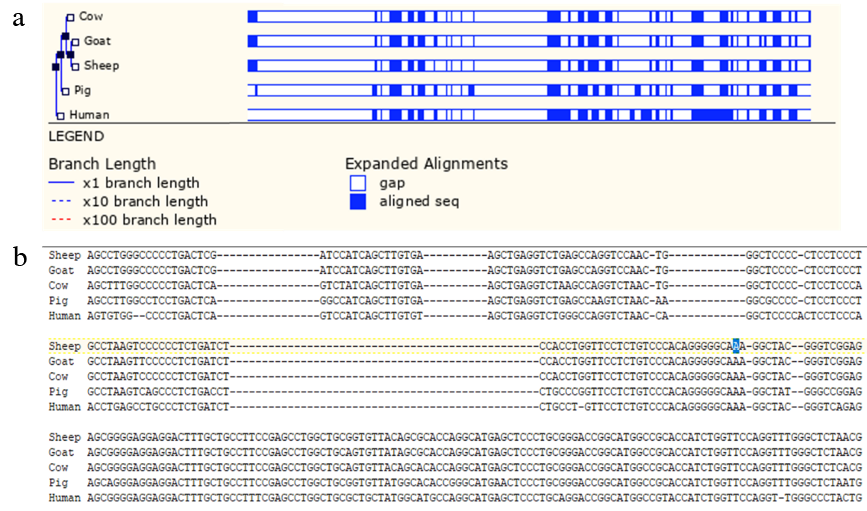


**Figure S3** Conservation Analysis of *NEIL1*. A: Comparison of the aligned regions of genomic sequences among species (Human, Pig, Cow, Goat and Sheep). B: Sequence alignment details near the edited sites of *NEIL1* (OAR18: 32339099, allele marked in blue).

**Table S1** Validation of RNA Editing Sites by Sanger sequencing. The chromatogram traces of gDNA and cDNA (extracted from kidney and spleen) are shown below.

| Position | Status | gDNA trace | cDNA (kidney) trace | cDNA(spleen) trace |
| --- | --- | --- | --- | --- |
| ABLIM1 (-)^1^  Chr22:33833292  C-to-A/G (+)^2^ | Common | 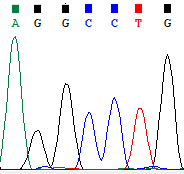 | 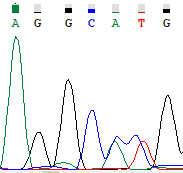 | 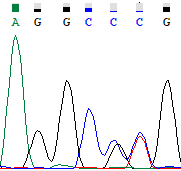 |
| ABLIM1 (-)  Chr22: 33833293  T-to-C (+) | Common |  |  |  |
| ABLIM1 (-)  Chr22:33833524  T-to-C (+) | Spleen specfic | 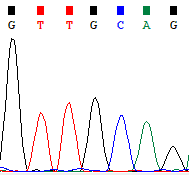 | 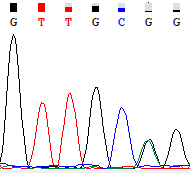 | 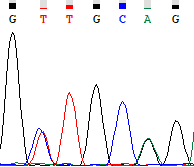 |
| ABLIM1 (-)  Chr22:33833528  A-to-G (+) | Common |  |  |  |
| DERA (-)  Chr3: 198476148  A-to-G (-) | Common | 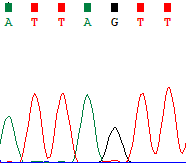 | 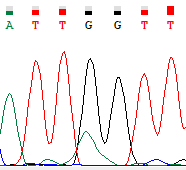 | 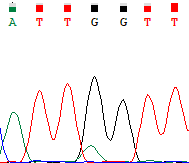 |
| DERA (-)  Chr3: 198476138  A-to-G (-) | Common | 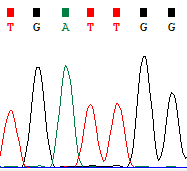 | 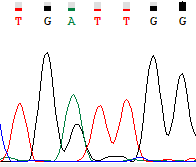 | 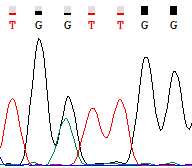 |
| AHNAK (-)  Chr21:  40171220  G (+) | Low editing  level | 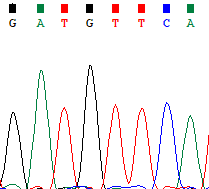 | 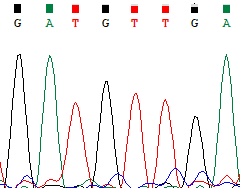 | 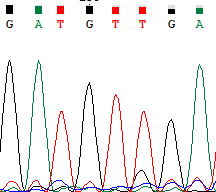 |
| AHNAK (-)  Chr21: 40171223  C-to-G (+) | Common |  |  |  |
| CMC1 (+)  Chr19:2652954  G (+) | Specfic | 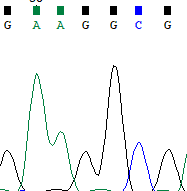 | 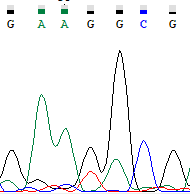 | 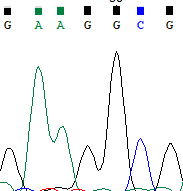 |
| GSN (-)  Chr2:2197292  G-to-A (-) | Common | 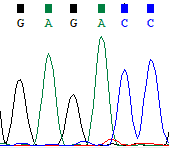 | 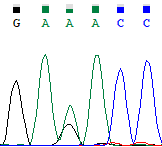 | 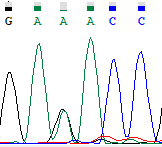 |
| STAT5B (-)  Chr11:41753132  C (-) | False positive | 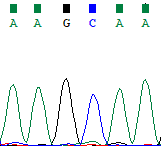 | 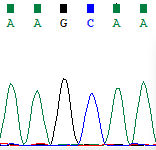 | 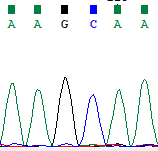 |
| CARD19 (-)  Chr2:28287028:C (-)  Chr2:28287030:C (-) | Not sure | Failed | 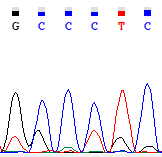 | 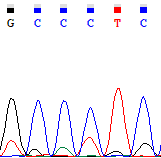 |

^1^ (-) = minus strand

^2^ (+) = plus strand

**Table S2** FPKM (mean ± SD) of *APOBEC3F* and *APOBEC2* in Kidney and Spleen

|  | Kidney | Spleen |
| --- | --- | --- |
| APOBEC3F | 2.59 ± 0.9 | 12.64 ± 1.76 |
| APOBEC2 | 0.02 ± 0.04 | 0.35 ± 0.21 |

**Table S3** PCR primers Used in Sanger Sequencing.

| Gene | F/R^1^ | Primer sequences (5’-3’) | Template type |
| --- | --- | --- | --- |
| *ABLIM1* | F | AAGTTGGGTCCTAGCAAACAGT | cDNA & DNA |
| *ABLIM1* | R | ATTAGCGTTCACCTGTGACCTT | cDNA & DNA |
| *AHNAK* | F | ACATAGACGTCTCCGGACCTAA | cDNA |
| *AHNAK* | R | GGTTTTGAAGTGGACATCAGGC | cDNA |
| *AHNAK* | F | GCCTTCTACCTTAGGCAGTGAA | DNA |
| *AHNAK* | R | CAAGGTGGACATAGACACTCCC | DNA |
| *CARD19* | F | CATGCCTGCCTTCACTTC | cDNA & DNA |
| *CARD19* | R | CGTGCCTGTCAATGATGA | cDNA & DNA |
| *CMC1* | F | ATTCTGGAGTCCTTATGGTAG | cDNA & DNA |
| *CMC1* | R | CCTGTTTGATTTGGTGTTTG | cDNA & DNA |
| *DERA* | F | TTGCCTCATTCTGGAGTATT | cDNA & DNA |
| *DERA* | R | GCTAAGGTCACTAAGTATTCTG | cDNA & DNA |
| *GSN* | F | TGAGGACACAGCCAAGGA | cDNA & DNA |
| *GSN* | R | TGTCACCGCCGTAGAACT | cDNA & DNA |
| *STAT5B* | F | CATCACCATTGCCTGGAAGT | cDNA & DNA |
| *STAT5B* | R | GAAGTCACCATCGTTGTCAAG | cDNA & DNA |

^1^F/R = forward primer/reverse primer

**Table S4** Nonsynonymous Common Editing Sites in Sheep Kidney of Sheep.

| Chr | Pos | Ref | Alt | Gene.refGene | ExonicFunc.refGene | cDNAChange | AAChange^1^ |
| --- | --- | --- | --- | --- | --- | --- | --- |
| NC_019458.2 | 16788255 | G | A | *PPCS* | nonsynonymous SNV | c^2^.G631A | p^3^.E211K |
| NC_019460.2 | 383688 | G | T | *ARRDC1* | stopgain | c.C2232A | p.C744X |
| NC_019460.2 | 99618871 | A | C | *MAP4K4* | nonsynonymous SNV | c.T2899G | p.S967A |
| NC_019460.2 | 99618884 | T | G | *MAP4K4* | nonsynonymous SNV | c.A2886C | p.R962S |
| NC_019460.2 | 209867962 | G | A | *PARP11* | nonsynonymous SNV | c.G85A | p.V29I |
| NC_019461.2 | 68860307 | A | C | *LOC105606599* | nonsynonymous SNV | c.A568C | p.N190H |
| NC_019461.2 | 111634288 | A | G | *LOC101107593* | nonsynonymous SNV | c.A511G | p.M171V |
| NC_019461.2 | 112412697 | C | T | *LOC101123290* | nonsynonymous SNV | c.G856A | p.G286R |
| NC_019461.2 | 112412801 | A | T | *LOC101123290* | nonsynonymous SNV | c.T752A | p.M251K |
| NC_019461.2 | 112413177 | A | G | *LOC101123290* | nonsynonymous SNV | c.T376C | p.Y126H |
| NC_019461.2 | 112413319 | G | T | *LOC101123290* | stopgain | c.C234A | p.C78X |
| NC_019461.2 | 112413321 | A | G | *LOC101123290* | nonsynonymous SNV | c.T232C | p.C78R |
| NC_019461.2 | 112536463 | C | T | *LOC105601949* | nonsynonymous SNV | c.G322A | p.D108N |
| NC_019461.2 | 112536469 | T | C | *LOC105601949* | nonsynonymous SNV | c.A316G | p.M106V |
| NC_019461.2 | 112595316 | C | G | *LOC105614177* | nonsynonymous SNV | c.C376G | p.Q126E |
| NC_019461.2 | 112595322 | T | A | *LOC105614177* | nonsynonymous SNV | c.T382A | p.Y128N |
| NC_019461.2 | 112595332 | C | A | *LOC105614177* | nonsynonymous SNV | c.C392A | p.A131E |
| NC_019462.2 | 19625342 | T | G | *SLC22A4* | nonsynonymous SNV | c.A109C | p.M37L |
| NC_019463.2 | 84518983 | G | A | *LOC101123042* | nonsynonymous SNV | c.C791T | p.A264V |
| NC_019463.2 | 84518995 | G | A | *LOC101123042* | nonsynonymous SNV | c.C779T | p.A260V |
| NC_019464.2 | 81847292 | C | G | *LOC101103726* | nonsynonymous SNV | c.C349G | p.Q117E |
| NC_019464.2 | 81847310 | G | A | *LOC101103726* | nonsynonymous SNV | c.G367A | p.A123T |
| NC_019466.2 | 29512571 | A | G | *LOC101109545* | nonsynonymous SNV | c.T397C | p.C133R |
| NC_019466.2 | 29512574 | C | T | *LOC101109545* | nonsynonymous SNV | c.G394A | p.A132T |
| NC_019467.2 | 15971000 | A | G | *COG3* | nonsynonymous SNV | c.A1906G | p.I636V |
| NC_019470.2 | 65916359 | T | C | *BLCAP* | nonsynonymous SNV | c.A5G | p.Y2C |
| NC_019470.2 | 79225791 | C | G | *LOC101120007* | nonsynonymous SNV | c.G465C | p.K155N |
| NC_019470.2 | 79225810 | A | G | *LOC101120007* | nonsynonymous SNV | c.T446C | p.V149A |
| NC_019470.2 | 79225832 | G | T | *LOC101120007* | nonsynonymous SNV | c.C424A | p.H142N |
| NC_019471.2 | 46563724 | T | C | *LOC105616883* | nonsynonymous SNV | c.A82G | p.T28A |
| NC_019475.2 | 32339099 | A | G | *NEIL1* | nonsynonymous SNV | c.A851G | p.K284R |
| NC_019477.2 | 27498064 | G | A | *LOC101108171* | nonsynonymous SNV | c.C989T | p.T330I |
| NC_019477.2 | 27498065 | T | A | *LOC101108171* | nonsynonymous SNV | c.A988T | p.T330S |
| NC_019477.2 | 28008590 | C | T | *LOC101113705* | nonsynonymous SNV | c.G448A | p.G150S |
| NC_019477.2 | 49312892 | A | T | *LOC101122734* | nonsynonymous SNV | c.A1156T | p.T386S |
| NC_019478.2 | 40171223 | C | T | *AHNAK* | nonsynonymous SNV | c.G8583A | p.M2861I |
| NC_019478.2 | 40175989 | T | C | *AHNAK* | nonsynonymous SNV | c.A3817G | p.M1273V |
| NC_019480.2 | 43122117 | A | G | *TUBB6* | nonsynonymous SNV | c.A292G | p.M98V |
| NC_019480.2 | 43122445 | G | T | *TUBB6* | nonsynonymous SNV | c.G619T | p.A207S |
| NC_019480.2 | 43122505 | A | G | *TUBB6* | nonsynonymous SNV | c.A679G | p.I227V |
| NC_019480.2 | 43122507 | C | G | *TUBB6* | nonsynonymous SNV | c.C681G | p.I227M |
| NC_019480.2 | 43122518 | G | C | *TUBB6* | nonsynonymous SNV | c.G692C | p.R231P |
| NC_019484.2 | 77076269 | G | C | *PLXNA3* | nonsynonymous SNV | c.C5134G | p.P1712A |
| NC_019484.2 | 77076274 | C | T | *PLXNA3* | nonsynonymous SNV | c.G5129A | p.S1710N |
| NC_019484.2 | 77076275 | T | G | *PLXNA3* | nonsynonymous SNV | c.A5128C | p.S1710R |

^1^AAchange = amino acid change

^2^c = cDNA change

^3^p = protein change

**Table S5** Nonsynonymous Common Editing Sites in the Spleen of sheep.

| Chr | Pos | Ref | Alt | Gene.refGene | ExonicFunc.refGene | cDNAChange | AAChange^1^ |
| --- | --- | --- | --- | --- | --- | --- | --- |
| NC_019458.2 | 16788234 | G | A | *PPCS* | nonsynonymous SNV | c^2^.G610A | p^3^.V204M |
| NC_019458.2 | 16788255 | G | A | *PPCS* | nonsynonymous SNV | c.G631A | p.E211K |
| NC_019458.2 | 154551895 | T | C | *ZNF654* | nonsynonymous SNV | c.T2374C | p.S792P |
| NC_019458.2 | 154551938 | C | T | *ZNF654* | nonsynonymous SNV | c.C2417T | p.A806V |
| NC_019458.2 | 187480987 | C | G | *LOC101121396* | nonsynonymous SNV | c.G1019C | p.S340T |
| NC_019458.2 | 266216902 | C | A | *LOC101114663* | nonsynonymous SNV | c.C14A | p.T5N |
| NC_019460.2 | 383688 | G | T | *ARRDC1* | stopgain | c.C2232A | p.C744X |
| NC_019460.2 | 179161641 | T | C | *LOC101105816* | nonsynonymous SNV | c.A412G | p.I138V |
| NC_019460.2 | 179161748 | C | A | *LOC101105816* | nonsynonymous SNV | c.G305T | p.G102V |
| NC_019460.2 | 179161769 | C | T | *LOC101105816* | nonsynonymous SNV | c.G284A | p.R95Q |
| NC_019460.2 | 179161799 | T | C | *LOC101105816* | nonsynonymous SNV | c.A254G | p.Q85R |
| NC_019460.2 | 209867962 | G | A | *PARP11* | nonsynonymous SNV | c.G85A | p.V29I |
| NC_019461.2 | 104631755 | C | A | *KIAA1147* | stopgain | c.G745T | p.E249X |
| NC_019461.2 | 111634288 | A | G | *LOC101107593* | nonsynonymous SNV | c.A511G | p.M171V |
| NC_019461.2 | 112412683 | C | T | *LOC101123290* | nonsynonymous SNV | c.G870A | p.M290I |
| NC_019461.2 | 112412697 | C | T | *LOC101123290* | nonsynonymous SNV | c.G856A | p.G286R |
| NC_019461.2 | 112412712 | T | A | *LOC101123290* | stopgain | c.A841T | p.K281X |
| NC_019461.2 | 112412717 | A | G | *LOC101123290* | nonsynonymous SNV | c.T836C | p.M279T |
| NC_019461.2 | 112412721 | C | T | *LOC101123290* | nonsynonymous SNV | c.G832A | p.D278N |
| NC_019461.2 | 112412801 | A | T | *LOC101123290* | nonsynonymous SNV | c.T752A | p.M251K |
| NC_019461.2 | 112413120 | A | C | *LOC101123290* | nonsynonymous SNV | c.T433G | p.F145V |
| NC_019461.2 | 112413133 | C | A | *LOC101123290* | nonsynonymous SNV | c.G420T | p.Q140H |
| NC_019461.2 | 112413177 | A | G | *LOC101123290* | nonsynonymous SNV | c.T376C | p.Y126H |
| NC_019461.2 | 112413319 | G | T | *LOC101123290* | stopgain | c.C234A | p.C78X |
| NC_019461.2 | 112413321 | A | G | *LOC101123290* | nonsynonymous SNV | c.T232C | p.C78R |
| NC_019461.2 | 112445002 | C | G | *LOC105615175* | nonsynonymous SNV | c.G192C | p.R64S |
| NC_019462.2 | 14748709 | C | G | *MYO1F* | nonsynonymous SNV | c.G2993C | p.R998T |
| NC_019463.2 | 72136758 | T | C | *LOC101110593* | nonsynonymous SNV | c.T1850C | p.M617T |
| NC_019463.2 | 72136772 | T | C | *LOC101110593* | nonsynonymous SNV | c.T1864C | p.S622P |
| NC_019466.2 | 13827174 | G | C | *EPPK1* | nonsynonymous SNV | c.G8185C | p.E2729Q |
| NC_019467.2 | 15971000 | A | G | *COG3* | nonsynonymous SNV | c.A1906G | p.I636V |
| NC_019468.2 | 55749076 | A | G | *LOC101103909* | nonsynonymous SNV | c.A140G | p.Y47C |
| NC_019468.2 | 55749077 | T | G | *LOC101103909* | stopgain | c.T141G | p.Y47X |
| NC_019470.2 | 16539730 | G | T | *LOC101109728* | nonsynonymous SNV | c.C3583A | p.R1195S |
| NC_019470.2 | 65916359 | T | C | *BLCAP* | nonsynonymous SNV | c.A5G | p.Y2C |
| NC_019471.2 | 23415323 | A | G | *LOC101116756* | nonsynonymous SNV | c.A620G | p.N207S |
| NC_019471.2 | 50391231 | C | G | *LOC101104776* | nonsynonymous SNV | c.G565C | p.E189Q |
| NC_019471.2 | 50394015 | A | G | *LOC101104776* | nonsynonymous SNV | c.T281C | p.V94A |
| NC_019471.2 | 52285769 | C | T | *DMPK* | nonsynonymous SNV | c.G308A | p.R103H |
| NC_019471.2 | 52285785 | A | T | *DMPK* | nonsynonymous SNV | c.T292A | p.S98T |
| NC_019471.2 | 52285788 | G | C | *DMPK* | nonsynonymous SNV | c.C289G | p.P97A |
| NC_019471.2 | 52285790 | G | C | *DMPK* | nonsynonymous SNV | c.C287G | p.S96C |
| NC_019471.2 | 59961121 | A | C | *LOC105601911* | nonsynonymous SNV | c.T511G | p.S171A |
| NC_019474.2 | 71169409 | C | G | *LOC101114469* | nonsynonymous SNV | c.G1463C | p.S488T |
| NC_019474.2 | 71169425 | G | A | *LOC101114469* | nonsynonymous SNV | c.C1447T | p.P483S |
| NC_019474.2 | 71169426 | G | C | *LOC101114469* | nonsynonymous SNV | c.C1446G | p.N482K |
| NC_019475.2 | 32339099 | A | G | *NEIL1* | nonsynonymous SNV | c.A851G | p.K284R |
| NC_019475.2 | 33702110 | G | A | *LOC101122311* | nonsynonymous SNV | c.C350T | p.T117I |
| NC_019477.2 | 27498064 | G | A | *LOC101108171* | nonsynonymous SNV | c.C989T | p.T330I |
| NC_019477.2 | 27498065 | T | A | *LOC101108171* | nonsynonymous SNV | c.A988T | p.T330S |
| NC_019477.2 | 28008590 | C | T | *LOC101113705* | nonsynonymous SNV | c.G448A | p.G150S |
| NC_019477.2 | 49274909 | G | C | *LOC101122475* | nonsynonymous SNV | c.G386C | p.C129S |
| NC_019477.2 | 49274910 | C | G | *LOC101122475* | nonsynonymous SNV | c.C387G | p.C129W |
| NC_019477.2 | 49312892 | A | T | *LOC101122734* | nonsynonymous SNV | c.A1156T | p.T386S |
| NC_019478.2 | 40175989 | T | C | *AHNAK* | nonsynonymous SNV | c.A3817G | p.M1273V |
| NC_019480.2 | 43122117 | A | G | *TUBB6* | nonsynonymous SNV | c.A292G | p.M98V |
| NC_019480.2 | 43122505 | A | G | *TUBB6* | nonsynonymous SNV | c.A679G | p.I227V |
| NC_019480.2 | 43122507 | C | G | *TUBB6* | nonsynonymous SNV | c.C681G | p.I227M |
| NC_019480.2 | 43122518 | G | C | *TUBB6* | nonsynonymous SNV | c.G692C | p.R231P |
| NC_019480.2 | 43122715 | A | T | *TUBB6* | nonsynonymous SNV | c.A889T | p.T297S |
| NC_019480.2 | 43122752 | A | T | *TUBB6* | nonsynonymous SNV | c.A926T | p.Y309F |
| NC_019481.2 | 1720530 | G | T | *PGP* | nonsynonymous SNV | c.C595A | p.P199T |
| NC_019484.2 | 77205202 | A | G | *FLNA* | nonsynonymous SNV | c.A5765G | p.Q1922R |
| NC_019484.2 | 77859729 | C | G | *HAUS7* | nonsynonymous SNV | c.C859G | p.Q287E |

^1^AAchange = amino acid change

^2^c = cDNA change

^3^p = protein change

**Table S6** Functional Annotation Results of RNA Editing Genes in the Kidney of Sheep.

| Category | Term | Count | PValue | Genes | Benjamini^1^ |
| --- | --- | --- | --- | --- | --- |
| GOTERM_CC_DIRECT | GO:0005777~peroxisome | 5 | 4.82E-04 | *HSDL2, HAO2, IDH2, PMVK, LOC101103726* | 0.04664091 |
| GOTERM_BP_DIRECT | GO:0007017~microtubule-based process | 3 | 0.01445988 | *LOC101122734, TUBB6, TUBB4A* | 0.98921773 |
| GOTERM_MF_DIRECT | GO:0005200~structural constituent of cytoskeleton | 3 | 0.02823229 | *LOC101122734, TUBB6, TUBB4A* | 0.95836742 |
| GOTERM_MF_DIRECT | GO:0015238~drug transmembrane transporter activity | 2 | 0.03351866 | *SLC17A3, SLC47A2* | 0.84925683 |
| GOTERM_CC_DIRECT | GO:0005829~cytosol | 11 | 0.04106427 | *PANK2, MKLN1, CLIC4, RAB29, ACMSD, IDH2, PMVK, TRAF6, LARP4B, AHNAK, DMPK* | 0.87451832 |
| GOTERM_MF_DIRECT | GO:0008565~protein transporter activity | 3 | 0.05352711 | *COG3, RAMP3, AP4B1* | 0.8693821 |
| GOTERM_BP_DIRECT | GO:0015937~coenzyme A biosynthetic process | 2 | 0.06716893 | *PANK2, PPCS* | 0.99997985 |
| GOTERM_BP_DIRECT | GO:0007020~microtubule nucleation | 2 | 0.07435129 | *SLAIN2, AKAP9* | 0.99966765 |
| GOTERM_BP_DIRECT | GO:0010596~negative regulation of endothelial cell migration | 2 | 0.08147902 | *APOH, NR2F2* | 0.99865049 |
| GOTERM_BP_DIRECT | GO:0006998~nuclear envelope organization | 2 | 0.08147902 | *PARP11, DMPK* | 0.99865049 |
| GOTERM_MF_DIRECT | GO:0008289~lipid binding | 3 | 0.08675475 | *PPARA, LOC101105816, STARD4* | 0.91940633 |
| GOTERM_MF_DIRECT | GO:0004857~enzyme inhibitor activity | 2 | 0.08952424 | *APBA3, CCAR2* | 0.87533106 |
| GOTERM_CC_DIRECT | GO:0048471~perinuclear region of cytoplasm | 6 | 0.09189232 | *NOX4, SEC31A, SLC17A3, CLIC4, APBA3, TRAF6* | 0.95845369 |
| GOTERM_BP_DIRECT | GO:0006641~triglyceride metabolic process | 2 | 0.09557223 | *SLC22A4, APOH* | 0.998066 |

^1^Adjusted P value by Benjamini method

**Table S7** Functional Annotation Results of RNA Editing Genes in the Spleen of Sheep.

| Category | Term | Count | PValue | Genes | Benjamini^1^ |
| --- | --- | --- | --- | --- | --- |
| GOTERM_BP_DIRECT | GO:0007017~microtubule-based process | 5 | 1.32E-04 | *LOC101119713, LOC101121396, LOC101122734, TUBB6, TUBB4A* | 0.06104461 |
| GOTERM_MF_DIRECT | GO:0005200~structural constituent of cytoskeleton | 5 | 4.17E-04 | *LOC101119713, LOC101121396, LOC101122734, TUBB6, TUBB4A* | 0.05551708 |
| GOTERM_MF_DIRECT | GO:0003924~GTPase activity | 7 | 0.00538108 | *LOC101119713, LOC101121396, GNA15, LOC101122734, TUBB6, LRRK2, TUBB4A* | 0.30898881 |
| GOTERM_MF_DIRECT | GO:0008565~protein transporter activity | 4 | 0.01406652 | *COG3, RAMP3, XPO5, AP4B1* | 0.47635007 |
| GOTERM_MF_DIRECT | GO:0001948~glycoprotein binding | 4 | 0.01406652 | *LCK, FBXO6, LRRK2, FLNA* | 0.47635007 |
| GOTERM_MF_DIRECT | GO:0016491~oxidoreductase activity | 5 | 0.02013437 | *PTGR2, PYROXD1, MAOA, CREG1, LOC101114663* | 0.50174286 |
| GOTERM_BP_DIRECT | GO:0006629~lipid metabolic process | 4 | 0.02683728 | *LIPG, SRD5A3, PLPP1, PNPLA6* | 0.99847864 |
| GOTERM_BP_DIRECT | GO:0045184~establishment of protein localization | 3 | 0.03373765 | *WDPCP, DLG4, FLNA* | 0.99573309 |
| GOTERM_MF_DIRECT | GO:0004888~transmembrane signaling receptor activity | 3 | 0.04938937 | *CD3G, CD3D, TLR2* | 0.75038366 |
| GOTERM_MF_DIRECT | GO:0003676~nucleic acid binding | 11 | 0.05525366 | *PAN2, ZNF93, IKZF3, LOC101122457, LOC105610483, RNASE6, LOC101107891, LOC101107593, ZNF654, ZNF333, RBM26* | 0.72687333 |
| GOTERM_CC_DIRECT | GO:0042105~alpha-beta T cell receptor complex | 2 | 0.05534346 | *CD3G, CD3D* | 0.9998955 |
| GOTERM_BP_DIRECT | GO:0032729~positive regulation of interferon-gamma production | 3 | 0.0608788 | *IL12RB2, IL18R1, IFNAR1* | 0.99944146 |
| GOTERM_MF_DIRECT | GO:0005525~GTP binding | 8 | 0.06103578 | *LOC101119713, LOC101121396, GNA15, LOC101109643, LOC101122734, TUBB6, LRRK2, TUBB4A* | 0.70845694 |
| GOTERM_BP_DIRECT | GO:0006000~fructose metabolic process | 2 | 0.06515849 | *PFKFB2, ALDOB* | 0.99838414 |
| GOTERM_BP_DIRECT | GO:0033089~positive regulation of T cell differentiation in thymus | 2 | 0.06515849 | *TESPA1, ADA* | 0.99838414 |
| GOTERM_BP_DIRECT | GO:0050862~positive regulation of T cell receptor signaling pathway | 2 | 0.06515849 | *TESPA1, ADA* | 0.99838414 |
| GOTERM_BP_DIRECT | GO:0006355~regulation of transcription, DNA-templated | 8 | 0.06605334 | *ZNF93, LOC101122457, TFEC, LOC105610483, CREG1, LOC101107891, LOC101107593, ZNF333* | 0.99562876 |
| GOTERM_BP_DIRECT | GO:0001921~positive regulation of receptor recycling | 2 | 0.07560119 | *RAMP3, SCRIB* | 0.99528415 |
| GOTERM_CC_DIRECT | GO:0005874~microtubule | 4 | 0.08423824 | *LOC101119713, LOC101122734, TUBB6, TUBB4A* | 0.99916152 |
| GOTERM_CC_DIRECT | GO:0032040~small-subunit processome | 2 | 0.08707781 | *LOC101112168, FCF1* | 0.99247317 |
| GOTERM_BP_DIRECT | GO:0015937~coenzyme A biosynthetic process | 2 | 0.09614083 | *PANK2, PPCS* | 0.99758727 |
| GOTERM_BP_DIRECT | GO:0016337~single organismal cell-cell adhesion | 3 | 0.09662046 | *ICAM2, RAPGEF1, SCRIB* | 0.99541728 |

^1^Adjusted P value by Benjamini method
